# Circulating Irisin Levels and Redox Status Markers in Patients with Gastric Cancer: A Case-Control Study

**DOI:** 10.31557/APJCP.2020.21.10.2847

**Published:** 2020-10

**Authors:** Shabnam Shahidi, Jalal Hejazi, Minoosh Moghimi, Soheila Borji, Saeed Zabihian, Mojtaba Fathi

**Affiliations:** 1 *Student Research Center, Zanjan University of Medical Sciences, Zanjan, Iran. *; 2 *Department of Nutrition, School of Medicine, Zanjan University of Medical Sciences, Zanjan, Iran. *; 3 *Department of Hemato Oncology, Valiasr Hospital, Zanjan University of Medical Sciences, Zanjan, Iran. *; 4 *Department of Radiology, School of Medicine, Zanjan University of Medical Sciences, Zanjan, Iran. *; 5 *Ayatollah Mousavi Clinical and Educational Center, Zanjan University of Medical Sciences, Zanjan, Iran. *; 6 *Department of Biochemistry. School of Medicine, Zanjan University of Medical Sciences, Zanjan, Iran. *

**Keywords:** Irisin, malondialdehyde, oxidative stress, gastric cancer, superoxide dismutase

## Abstract

**Objective:**

Irisin, mostly known as an exercise-induced fat browning myokine, has been recently detected in several cancer cells, and its potential for being utilized as a biomarker for early diagnosis of some cancers, such as Gastric cancer (GC), is the subject of speculation. The present study aims to compare serum irisin levels in GC patients and healthy controls and assess the interrelation between irisin and oxidative stress markers.

**Methods:**

In this case-control study, 22 newly diagnosed GC patients and 29 healthy controls were recruited based on the inclusion criteria. Serum levels of irisin were quantified in duplicates by ELISA. Oxidative stress indices, including total antioxidant power in sera, thiol group, malondialdehyde, and superoxide dismutase concentrations, were also measured in both groups. An independent-sample t-test was used to compare the means between the two studied groups.

**Results:**

Serum levels of irisin were significantly higher in the GC group compared with those of their healthy counterparts (p =0.032). No significant differences were observed between the two groups in terms of the serum total antioxidant power or the oxidative stress marker, including MDA, thiol groups, and SOD concentration in sera. Furthermore, there was no significant association between irisin, FRAP, the Thiol group, and the SOD activity.

**Conclusion:**

According to the finding, the increased serum levels of irisin in GC patients can play a potential role in the early diagnosis of the GC patients; hence, this peptide can be employed as a new diagnostic indicator of GC.

## Introduction

Gastric cancer (GC), with a mortality rate of over 75%, is one of the two leading causes of cancer deaths and the fifth most common malignancy in the world (Sitarz et al., 2018). It is estimated that the 5-year survival of GC is approximately 30% among diagnosed cases; however, this survival rate can rise to 90% by timely diagnosis (Necula et al., 2019). This delay in diagnosis is due to the asymptomatic nature of the disease until it progresses to advanced stages. Moreover, upper gastrointestinal endoscopy, which is currently used for GC diagnosis, is an invasive procedure. Thus, new studies have focused on the discovery of less-invasive diagnostic; among them, serum indices have received the most attention (Necula et al., 2019). Unfortunately, the current gastrointestinal tumor biomarkers, such as carcinoembryonic antigen (CEA) and cancer antigen (CA) 72-4, are not very precise (Matsuoka and Yashiro, 2018). Hence the discovery of biomarkers with higher levels of sensitivity and specificity to screen process is imperative. 

Although irisin is mostly noted as an exercise-induced and fat browning myokine, lately, its potential role in affecting various medical conditions, such as cancer, has led to much debate and speculation. Irisin has been detected in many cancer cells, including those of breast, ovarian, lung, kidney, thyroid, and gastric; accordingly, some studies have suggested a diagnostic or even therapeutic role for this protein (Askari et al., 2018). However, the studies in this area, especially on humans, are far from exhaustive; also, some of these studies have yielded controversial results. For example, in an investigation by Provatopoulou et al., (2015), the investigators reported lower serum levels of irisin in breast cancer patients compared to those of the healthy controls, whereas Aydin et al., (2016) reported an increased level of irisin in gastrointestinal cancers.

On the other hand, reactive oxygen and nitrogen species (ROS/RNS) are considered as potential diagnostic and prognostic markers in many types and stages of cancers (Moldogazieva et al., 2018). In the stomach, one of the causes of the incidence of gastric ulcer, which predisposes to GC, is the increase in the adrenocortical activity following various factors, especially oxidative stress (Suzuki et al., 2011). Besides ulcerogenesis, oxidative stress is also responsible for the pathogenesis of gastric inflammation and carcinogenesis in helicobacter pylori infection, another GC predisposing factor (Suzuki et al., 2012). Different antioxidant systems, including superoxide dismutase (SOD), catalases, and the enzymes of the glutathione redox cycle, eliminate excessive ROSs under healthy conditions (Nathan and Cunningham-Bussel, 2013). As a result, any factor with the ability to boost the antioxidant capacity of the body can be considered as an anti-cancer agent. 

Several recent studies have proposed an antioxidant role for irisin. It seems irisin confers its antioxidant effects through different mechanisms. Increasing Glutathione peroxidase (GPX), catalase, and SOD activity and reducing malondialdehyde (MDA) levels are some of these proposed mechanisms (Askari et al., 2018). Considering all these issues and the want of studies, especially on humans, studying irisin levels and redox status in GC patients and comparing them with those of the normal subjects, together with investigating the interaction between irisin and oxidative status or antioxidant enzymes, seems to be a requirement, guiding us to find new diagnostic biomarkers. This study aims to investigate the irisin serum levels and redox status of GC patients and their possible interrelationship with one another. 

## Materials and Methods

*Study population*

In this case-control study, 22 newly diagnosed gastric cancer patients with adenocarcinoma pathology were recruited as the case group. The patients were selected from the oncology clinic of Valiasr hospital of Zanjan based on the inclusion criteria between September 2018 and April 2019. On the other hand, 40 volunteers referred to the Laboratory of Valiasr hospital for annual screening were assessed. Among them, 29 people without documented diseases and with normal blood test results were recruited as the control group. Eventually, informed consent was obtained from all 51 participants. 

 The inclusion criterion for cases was confirmation of gastric cancer by an endoscopic biopsy. On the other hand, any gastric surgery or resection, previous chemotherapy, distant metastasis, double-primary cancer, other severe illnesses, including diabetes or cardiovascular diseases, and being an athlete or having a regular exercise program, were defined as exclusion criteria. The study was approved by the ethics committee of Zanjan University of Medical Sciences with the ethical code IR.ZUMS.REC.1397.168.

*Biochemical measurements*

Peripheral venous blood samples were collected from all participants after an overnight fast lasted for at least 8 hours. Samples were immediately centrifuged at 3,000 rpm and normal temperature for 10 min. Then, the supernatant was taken and aliquoted into two microtubes, one for storing at -80°C until performing enzyme-linked immunosorbent assay (ELISA) and the other for measuring other variables, namely biochemical, by biochemical analyzer prestige (Biolis 50I, Tokyo, Japan). 

*Serum irisin measurement*

Serum irisin levels were quantified in duplicates using commercially available ELISA kits (Bioassay Technology Laboratory, China), with a sensitivity of 0.03 ng/ml. The intra- and inter-assay coefficients of variation to express the precision of immunoassay results were also10 % and 12%, respectively.

*Hematological measurements*

Hematological indices (hematocrit (HCT), red blood cells (RBC), white blood cells (WBC), and Plt count) were measured by an automated cell counter (Sysmex KX-21 cell counter, Kobe, Japan).

*Oxidative stress indices*

The MDA concentration was determined using the Thiobarbituric acid reactive substances (TBARS assay), following Buege and Aust procedure (Sanchez-Delgado et al., 2015). Total antioxidant power in sera of both groups was measured using the ferric reducing antioxidant power (FRAP) assay, following Benzie and Strain (1996) protocols. The activity of SOD (EC.1.15.1.1), which converts superoxide radicals to hydrogen peroxide and molecular oxygen, was determined based on the protocol of Wintburn et al., (1975). Serum thiol groups were evaluated according to the protocol of Hu (1994) using a spectrophotometric assay. 

*Anthropometric measurements*

Anthropometric values, including height and weight, were measured in light clothing without shoes using the SECA stadiometer to the nearest 0.1 cm and SECA electronic scale to the nearest 0.05 kg, respectively. 

*Statistical analysis*

The quantitative variables were presented as mean and standard deviation or median and range, whereas the qualitative variables were reported as frequencies and percentages. Kolmogorov-Smirnov test was applied to assess the normality of the distribution of the variables. When the distribution was normal, an independent-sample t-test was used to compare the means between the two studied groups; in the cases of not normally distributed data, the Mann Whitney U test was applied. All tests were two-tailed, and p-values <0.05 were considered as statistically significant. For statistical analysis of data, SPSS Version 20 (IBM Corporation, Armonk, NY, USA) was applied. 

## Results

A total of 51 participants were enrolled in the study (22 cases and 29 controls). The characteristics of each study group are presented in Table 1. As observed, there were no significant differences between the two groups in terms of age, sex, as well as biochemical and hematological parameters. However, weight and BMI in the GC group were significantly lower than those of the control. 

Table 2 presents serum irisin level and oxidative stress markers in gastric cancer patients and the healthy control group. As seen, the serum irisin level was significantly higher in the GC group compared with that of their healthy counterparts. However, no significant differences were observed between the two groups in terms of the serum total antioxidant power or the marker of oxidative stress, including MDA, thiol groups, and SOD concentration in sera (Table 2). 

According to the exploratory correlation analysis, there were no significant correlations between irisin, the FRAP, the Thiol group, and SOD activity. 

**Table 1:** Characteristics of the Studied Groups

	Gastric cancer Patients (n=22)	Healthy Controls (n=29)	P
Sex (male)	17 (77.3%)	16 (55.2%)	0.105
Age (y)	65.82±12.300	56.17±17.536	0.073
Height	168.48±8.612	165.60±10.388	0.488
Weight	61.429±9.9578	69.607±14.6378	0.032
BMI (kg/m^2^)	21.629±3.0645	25.798±4.1088	<0.001
Systolic pressure (mmHg)	11.364±.8888	11.554±1.0571	0.428
Diastolic pressure (mmHg)	6.773±1.0771	7.696±1.3900	0.11
BS(mg/dl)	99.95±18.282	96.05±11.592	0.389
BUN(mg/dl)	17.259±6.7276	15.419±7.1429	0.471
Cr (mg/dl	0.926±.1870	0.963±.2395	0.538
Na (mEq/L)	138.991±3.8916	142.237±3.5208	0.818
K (mEq/L)	4.245±.4021	4.106±.4293	0.602
RBC ( ×10^6^μl^-1^)	4.5±0.72	4.7±0.55	0.359
HCT (%)	35.85 (16.7-41.6)	39.65 (30.9-47.4)	0.002
WBC ( ×10^3^μl^-1^)	6.0 (3.7-17.1)	6.50 (3.9-14.9)	0.599
Plt count	233.0 (95- 378)	200.0 (119-472)	0.145

**Table 2:** Serum Irisin Level and Oxidative Stress Markers in Cases and Controls

	Gastric cancer group (n=22)	Control group (n=29)	p
Irisin (ng/ml)	0.41900 (0.299-1.790)	0.35900 (0.028-0.678)	0.032
FRAP (μmol/l)	707.286 (57.3-1027.3)	707.286 (547.3-882.3)	0.455
Thiol group (μmol/l)	0.05046±0.015129	0.04592±.012420	0.25
MDA (nmol/ml)	2.061857± 0.608551	1.866141±0.443483	0.19
SOD (U/ml)	19.95042 (3.884-59.675)	29.13100 (1.412-86.158)	0.128

## Discussion

According to the results of the present study, the serum irisin level was significantly higher in GC patients. However, there were no significant differences between the two studied groups in terms of the total antioxidant power or the oxidative stress marker, including MDA, thiol groups, and SOD concentration in sera. Further, no significant relationship was observed between irisin and the oxidative stress markers. 

In line with the findings of the current research, several studies have documented increasing levels of irisin in different cancers. For instance, Altay et al., (2018) showed that patients with renal cancer had higher irisin levels compared to the control group. Aydin et al., (2016) confirmed an increased irisin level in the vast majority of gastrointestinal cancers, using immunohistochemistry (IHC). Increased expression pattern of irisin was also seen in different types of breast, ovary, and cervix carcinomas (Kuloglu et al., 2016). Moreover, in the recent study of Ugur et al., (2019) irisin was described as a potential immunohistochemical marker for identifying different types of thyroid cancers. However, there are still some controversies in this regard. As an instance, in the study of Gagini et al., (2017) on the mRNA level, a significant difference was observed between irisin in the liver of the patients with hepatocellular carcinoma (HCC) and that of the controls; however, irisin concentration in plasma was not significantly different between cancer and control groups. 

Elevation of the irisin levels in GC patients observed in the present study can be considered as the body’s defensive action against cancer progression. A possible mechanism in this regard can be the stimulation of mitochondria uncoupling protein-1 (UCP-1) expression by irisin, uncoupling the electron transport chain from the energy generation process by which the potential energy of food is released as heat-instead adenosine triphosphate (ATP) (Zhang et al., 2014). As cancer cells are not resistant to heat (Aydin et al.,, 2016), this local hyperthermia can have deleterious effects on the tumor niche by destructing nutrient blood vessels and coagulating the cancer cell proteins. It seems irisin inhibits epithelial-mesenchymal transition (EMT) and thePI3K/AKT/Snail signaling pathway, controlling the migration and invasive ability of cancer cells (Shao et al., 2017). 

Cachexia is a common condition in GC patients, especially in advanced stages. Elevated levels of irisin in GC patients can explain the high frequency of cachexia in these patients, at least in part. Irisin inhibits de novo lipogenesis (DNL), as a compensatory response to induced lipogenesis during the cancer progression (Gaggini et al., 2017), and also hydrolysis triacylglycerols (TAGs) in white adipose tissue (Us Altay et al., 2016). All of these events are responsible for weight loss during cancer. Thus, the negative correlation between serum irisin levels and BMI in some studies (Moreno-Navarrete et al., 2013), as well as the present study (r= -0.404, p<0.01), is expected.

Some recent studies have demonstrated that the overproduction of ROSs is the primary cause of many cancers, such as GC (Davalli et al., 2018). However, there are still controversies regarding the bodily activity of the antioxidant system, including GPX, Catalase, and SOD, in cancer patients. As an example, while several studies have reported increased SOD activities in GC patients compared to those of the healthy controls (Malafa et al., 2000), some studies have shown a reduction in the SOD activity (Lan-kai-wei, 1991). Such controversies also exist on other oxidative stress markers in different research. In the present study, no significant difference is observed in redox status markers between the two studied groups. The existent controversies in this regard can be justified if the studied populations are considered to be in different stages of cancer.

For the first time, Zhu et al., (2015) proposed an antioxidant role for irisin through suppressing the activation of PKC-β/ NADPH oxidase and NF-κB/iNOS pathways and the formation of peroxynitrite. Another study of diabetic vascular complications hinted at an irisin’s inhibitory effect on ROS-NLRP3 inflammasome signaling; this action occurs through the mitigation of advanced glycation end products-induced inflammation (Deng et al., 2018). The antioxidant role of irisin in protecting cardiomyocytes has also been reported in several studies (Askari et al., 2018). Hence, in the present research, it is hypothesized that the controversial anti-cancer effects of the irisin, at least in part, could stem from its antioxidant effects and changes it impose in redox status. Indeed, increased irisin levels during gastric cancer could be a physiological response to oxidative stress. However, no significant relationship is observed between irisin levels and oxidative stress markers since most of the patients are in the early stages of cancer, and their irisin levels and even plasma levels of antioxidant enzymes are relatively low. Implementation of this study with patients in more advanced stages of the cancer is recommended for future studies. 

The current research confronted certain limitations. First, the study lacked the expression analysis of the irisin precursors: PGC1-α and FNDC5. Second, the molecular analysis able to assist in clarifying the reasons for the elevation of sera level of irisin in GC patients was not performed. Lastly and most importantly, the population size of the study groups was relatively small. Thus, the verification of the results by a future study with a larger number of subjects would be rewarding.

In conclusion, serum irisin levels of GC patients were significantly higher than those of the healthy controls in the present study; thus, there is a probability of using irisin as a biomarker for early detection of some cancers, such as gastric in non-athlete individuals. There were no significant differences between the two studied groups in terms of different oxidative stress markers. Further, there was no significant relationship between serum irisin level and oxidative stress markers (e.g., SOD and FRAP).
